# Open Design 3D-Printable Adjustable Micropipette that Meets the ISO Standard for Accuracy

**DOI:** 10.3390/mi9040191

**Published:** 2018-04-18

**Authors:** Martin D. Brennan, Fahad F. Bokhari, David T. Eddington

**Affiliations:** Department of Bioengineering, University of Illinois at Chicago, Chicago, IL 60607, USA; mbrenn3@uic.edu (M.D.B.); fbokha2@uic.edu (F.F.B.)

**Keywords:** open source labware, 3D printing, functional prototyping

## Abstract

Scientific communities are drawn to the open source model as an increasingly utilitarian method to produce and share work. Initially used as a means to develop freely-available software, open source projects have been applied to hardware including scientific tools. Increasing convenience of 3D printing has fueled the proliferation of open labware projects aiming to develop and share designs for scientific tools that can be produced in-house as inexpensive alternatives to commercial products. We present our design of a micropipette that is assembled from 3D-printable parts and some hardware that works by actuating a disposable syringe to a user-adjustable limit. Graduations on the syringe are used to accurately adjust the set point to the desired volume. Our open design printed micropipette is assessed in comparison with a commercial pipette and meets the ISO 8655 standards.

## 1. Introduction

The open source development model, initially applied to software, is thriving in the development of open source scientific equipment due in part to increasing access of 3D printing [1,2]. Additive manufacturing methods have existed for decades although the recent availability of inexpensive desktop printers [3,4] has made it feasible for consumers to design and print prototypes and even functional parts, as well as consumer goods [5,6]. The proliferation of free CAD software [7,8,9,10] and design sharing sites [11,12,13,14] has also supported the growth and popularity of open designed parts and projects. Open design 3D-printable lab equipment is an attractive idea because, like open source software, it allows free access to technology that is otherwise inaccessible due to proprietary and/or financial barriers. Open design tools create the opportunity for scientists and educational programs in remote or resource-limited areas to participate with inexpensive and easy to make tools [15,16,17,18,19,20]. Open source development also enables the development of custom solutions to meet unique applications not met by commercial products that are shared freely and are user modifiable [5,21,22,23,24,25,26]. Some advanced, noteworthy, open source scientific equipment include a PCR device [27], a tissue scaffold printer [28] and a two-photon microscope [29], although simple tools have the potential to be impactful as they can serve a wider community.

Some simple and clever printable parts that have emerged are ones that give a new function to a ubiquitous existing device, such as a drill bit attachment designed to hold centrifuge tubes, allowing a dremel to be used as a centrifuge [30]. Although this may make a rather crude centrifuge, it may be an adequate solution for a fraction of the cost of a commercial centrifuge. Other examples of open source research tools that utilize 3D-printed parts include optics equipment [31], microscopes [32,33], syringe pumps [34,35], reactionware [36,37,38,39] and microfluidics [40,41], and the list continues to grow [42].

One example of an everyday scientific tool that provides the opportunity for an open design solution is the micropipette. An open design micropipette that can be made inexpensively affords more options for labs and educational settings. Micropipettes are an indispensable tool used routinely in lab tasks and can easily cost $1000 USD for a set. Often, a lab will require several sets each for a dedicated task. Some pipettes may even be re-calibrated for use with liquids of different properties.

Air displacement pipettes use a piston operating principle to draw liquid into the pipette [43]. In a typical commercial pipette. the piston is made to be gas-tight with a gasketed plunger inside a smooth barrel. Consumer-grade fused deposition modeling (FDM) printers are unable to build a smooth surface due to the formation of ridges that occur as each layer is deposited [44]. The ridges formed by FDM make it impractical to form a gas-tight seal between moving parts, even with a gasket. Existing printable open-design micropipettes get around this limitation by stretching a membrane over one end of a printed tube, which when pressed causes the displacement. The displacement membrane can be made from any elastic material such as a latex glove. A few open design micropipettes exist including a popular one, which in addition to the printed parts, uses parts scavenged from a retractable pen [45]. A major limitation of this design is that there is no built-in feature such as a readout for the user to set the displacement to a desired volume. This requires the user to validate the volumes dispensed with a high precision scale. Without verification with a scale, the volumes dispensed can only be estimated based on the calculations of the deflection of the membrane, which is not a practical protocol.

We submit a new design whose major strength is the ability to adjust to any volume aided by the built-in scale. Our open design 3D-printable micropipette works by actuating a disposable syringe to a user-adjustable set point. This allows the user to set the pipette to a volume by reading the graduations on the syringe barrel. We have also designed an adjusted graduation scale, to be used in place of the printed on scale, that corrects for the compressibility of air, which allows the pipette to be set accurately. Additionally, our pipette offers a simplified assembly requiring no glue, tape or permanent connections.

## 2. Materials and Methods

**Figure 1:**
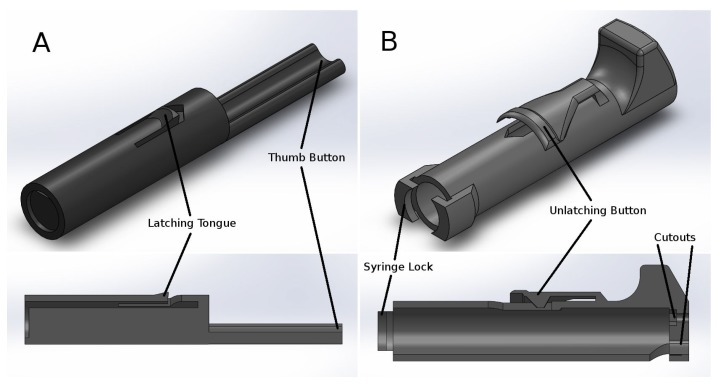
CAD renderings of printable parts and cross-sections. (**A**) The printed plunger part is a shaft that pushes on the syringe plunger and slides in the printed body part. The printed plunger has a latching tongue and button, which interfaces with the printed body part. (**B**) The printed body part holds the syringe and interfaces with the printed plunger part. The body part features the unlatching button and slots to hold the syringe in place by its flanges. The body part also has two cutouts in the top for the plunger button and for the hex nut and bolt. STL files of the printable parts are available in the Supplementary Materials.

Our printed pipette is designed to actuate a 1-mL or 3-mL syringe to a user-set displacement. The core of the design is two printed parts, the body and the plunger, which are able to be printed on a consumer-grade FDM printer (Figure 1), in this case a Makerbot Replicator.

**Figure 2:**
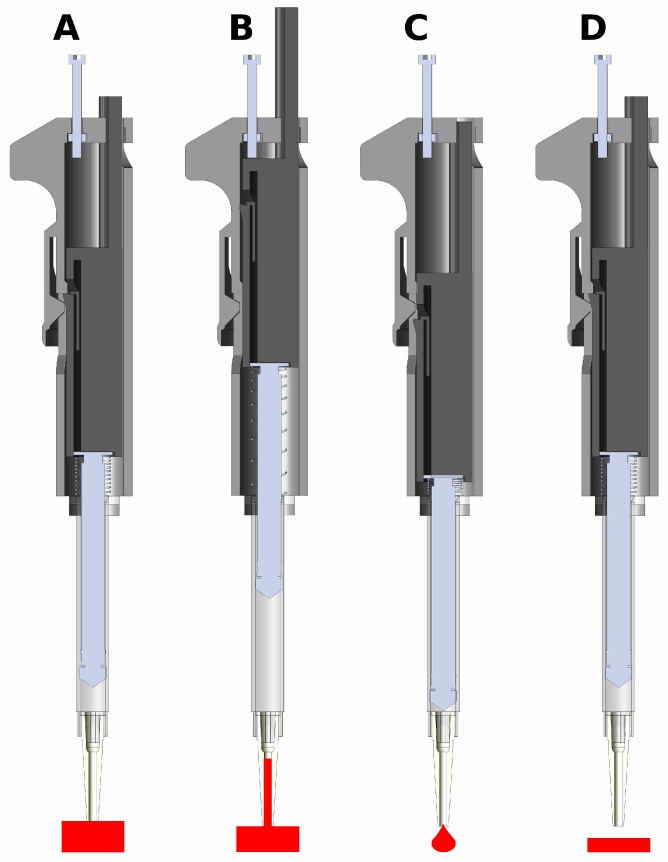
CAD renderings of assembled pipette and function. The pipette actuates the syringe to three positions. (**A**) The latched position. When the plunger is pressed, the pipette locks at this position. The tip is then placed in a liquid, and the unlatching button is pressed to release the pipette back to the set position (**B**), drawing in liquid. (**B**) The set position. The position of the screw determines the total displacement that the plunger moves. The pipette is spring loaded to return to this position. (**C**) The blow-out position. The fluid is transferred by pressing the plunger past the latched position to blow-out all the liquid. (**D**) Return to the latched position. The pipette returns to the latched position ready to perform another transfer.

A 1-mL or 3-mL syringe twists to lock in the body part and is held into place by the syringe flanges. The 30–300-μL configuration uses a 1-mL pipette, and the 100–1000-μL configuration uses a 3-mL pipette. The plunger part slides freely in the body part and actuates the syringe by pushing the thumb button (Figure 2). The pipette is spring loaded towards a set point, which is adjustable by a set-screw. When the thumb button is pressed, the system locks when it reaches the latched position, where it is ready to draw in fluid. The plunger is held in place with a latching mechanism, which when released, draws in fluid. The displacement is equal to the distance between the set position and the latched position. The pipette can also be pressed past the latched position to ‘blow-out’ the transferred fluid completely from the pipette tip. For a demonstration of the assembly and operation, see the videos in the Supplementary Materials. Additional materials required for assembly include two springs, a nut and a bolt (Table A1 and Table A2). Attempts to make a printable luer lock adapter for tips was abandoned as the surface of printed parts is too rough to make an air-tight seal with the luer or pipette tip. Instead, a combination of a barbed luer adapter and elastic tubing is used to attach the pipette tips. Our printed pipette mimics commercial pipettes in design, function and user operation, making it intuitive to use.

**Figure A1:**
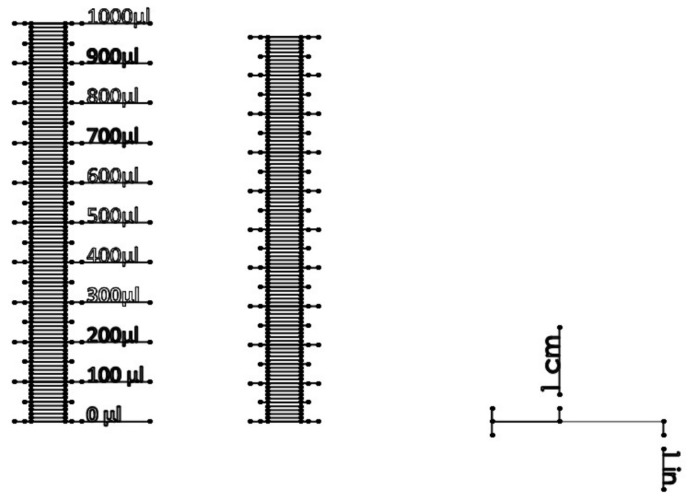
Adjusted scale graduations for the 30–300-μL pipette. Adjusted scale graduations that can be printed on paper or a transparency sheet and taped onto a 1-mL syringe. Using these graduations will correct for expansion of air and allow ISO standards to be met. One-inch and 1-cm marks to check the scale are included, as well as a reproduction of the existing 1-mL syringe graduation marks.

**Figure A2:**
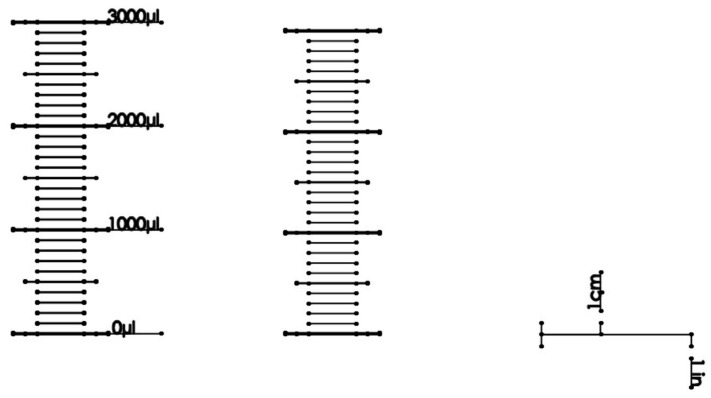
Adjusted scale graduations for the 100–1000-μL pipette. Adjusted scale graduations that can be printed on paper or a transparency sheet and taped onto a 3-mL syringe. Using these graduations will correct for expansion of air and allow ISO standards to be met. One-inch and 1-cm marks to check the scale are included, as well as a reproduction of the existing 3-mL syringe graduation marks. Print this image to 100% scale.

Our pipette uses the air-displacement method, where a vacuum is applied to a pocket of air to draw liquid into the pipette. As air is a compressible fluid, this pocket of air expands due to the weight of the liquid pulling on it. Due to this effect, the graduations on the syringe are not accurate, as they are designed for measuring liquid within the syringe. At larger volumes, this effect is more pronounced, resulting in the volume measured being greater than the amount of liquid pulled into the syringe. We remedied this by creating a new scale to account for the expansion. The scale is printed on a transparency sheet and is taped onto the syringe for accurate measurements (the scales appear in Appendix A: Figure A1 and Figure A2).

### 2.1. Fabrication and Assembly

**Figure 3:**
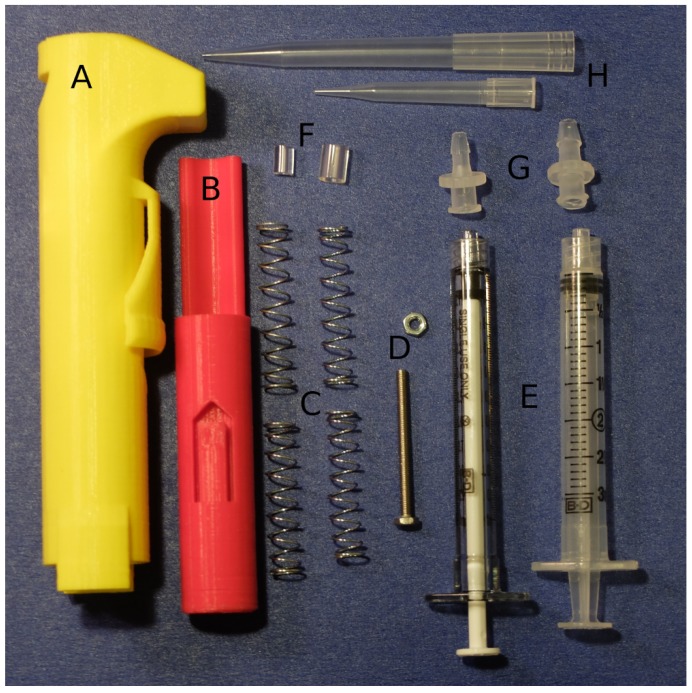
Photo of the parts to make the pipette. (**A**) Printed body part. (**B**) Printed plunger part. (**C**) Springs. (**D**) M3 hex nut and M3 bolt. (**E**) Disposable syringes. (**F**) Two sizes of tubing to adapt pipette tips to luer barbs. (**G**) Luer lock syringe-to-barb adapters. (**H**) Pipette tips. A list of parts is in Appendix A: Table A1 and Table A2.

**Figure 4:**
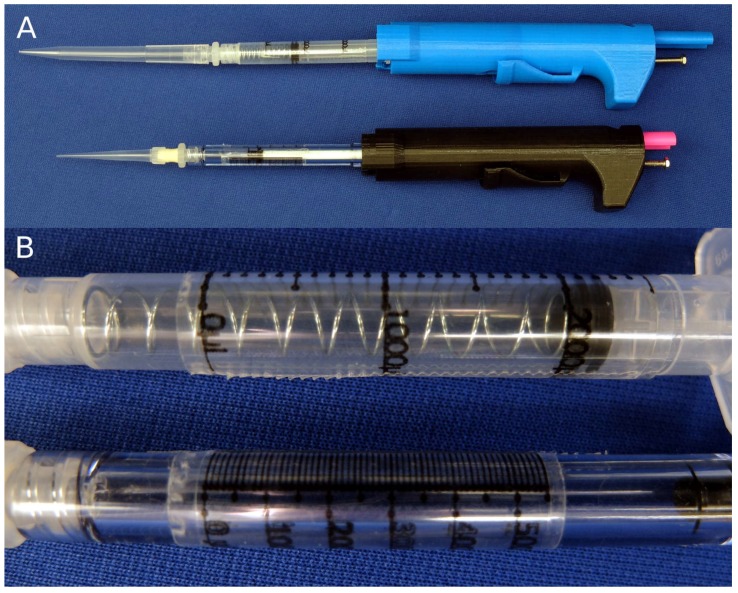
Photos of assembled pipettes. (**A**) Two assemblies of the pipette: the 100–1000-μL configuration (top) and the 30–300-μL configuration (bottom). (**B**) Close-up photo of the taped on scale for each of the syringes. Scales are available in the Appendix A: Figure A1 and Figure A2.

Two parts are printed at normal resolution with rafts on a Makerbot Replicator (STL files are available in the Supplementary Materials). A disposable syringe and a few extra parts are used to assemble the pipette (Figure 3). A small amount of paraffin wax is applied to the screw to prevent slop from causing the set point to drift after each actuation. The nut is sunk into the hex inset in the printed body part. The bolt is threaded in from the top of the body into the nut. Two springs are threaded onto the plunger of a 1-mL syringe for the 30–300-μL configuration. Springs are placed inside a 3-mL syringe for the 100–1000-μL configuration. The plunger part is inserted in the body, and the syringe assembly is pushed in and locked from the syringe flanges to the body part to complete assembly (Figure 4). Parts and cost are listed in Table A1 and Table A2 in Appendix A.

### 2.2. Validation

The printed pipette’s accuracy and precision were characterized and compared to a commercial pipette, as well as ISO 8655. The printed pipette was adjusted to the target volume by eye from the syringe graduations. Deionized water was transferred and measured with a high precision scale. Five transfers were recorded and averaged to account for random variability. Data were taken for printed pipettes with existing syringe graduations, as well as with our adjusted scale. Data were taken with commercial pipettes of 30–300 μL and 100–1000 μL to compare to the printed pipette. Accuracy and precision are expressed as systematic error and random error, respectively, and are calculated according to ISO 8655 [43] (Table 1 and Table 2). The systematic error, or accuracy, is calculated according to the following equations where accuracy (*A*) is the difference of the mean volume ($\bar{V}$) and the nominal volume ($V_{o}$):

(1) $$A=\bar{V}-V_{o},\;A\%=100\%\times A/V_{o}$$

The precision or random error is the standard deviation (*s*) of the measurements, and (cv) is the coefficient of variation:

(2) $$s=\sqrt{\frac{\sum_{i=1}^{n}{\left(V_{i}-\bar{V}\right)}^{2}}{n-1}},\;cv=100\%\times s/\bar{V}$$

## 3. Results

**Figure 5:**
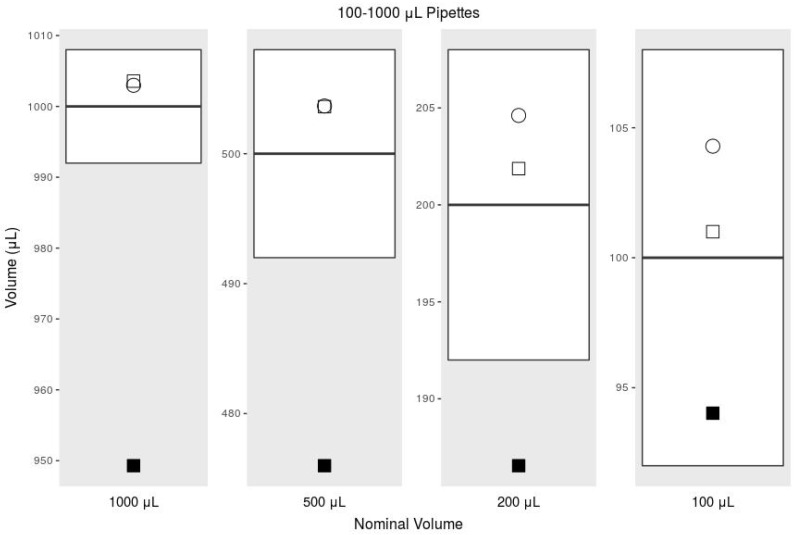
Comparison of the accuracy, or systematic error, among 100–1000-μL pipettes. The box plot indicates the range of the ISO standard; the plotted circle indicates the commercial pipette; and the plotted squares are the printed pipette. Empty squares are with the printed pipette with our adjusted graduation scale, and the filled squares are measurements taken with the syringe’s existing graduations (data from Table 1.)

**Figure 6:**
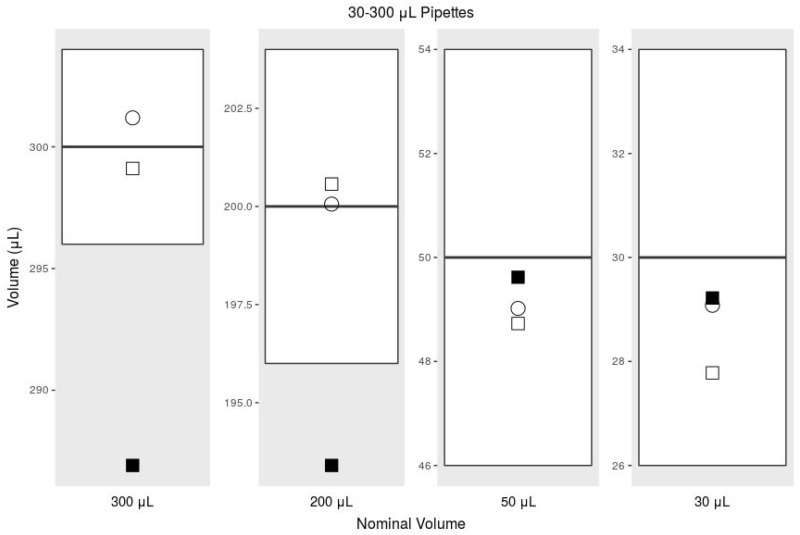
Comparison of the accuracy, or systematic error, among 30–300-μL pipettes. The box plot indicates the range of the ISO standard; the plotted circle indicates the commercial pipette; and the plotted squares are the printed pipette. Empty squares are with the printed pipette with our adjusted graduation scale, and the filled squares are measurements taken with the syringe’s existing graduations (data from Table 2.)

Using the adjusted syringe graduation scale, our printed pipette meets ISO 8655 standards, but using the existing syringe graduation scale does not meet the ISO standard for accuracy. According to ISO 8655 for a pipette with the maximum nominal volume of 1000 μL, the systematic error cannot exceed 8 μL, and the random error cannot exceed 3 μL [43]. For the maximum nominal volume of 300 μL, the systemic error cannot exceed 4 μL, and the random error cannot exceed 1.5 μL. The commercial pipettes met these standards handily, but in our initial tests with our printed pipette, we noticed large negative systematic error, suggesting that we were missing a biasing factor. This error was exaggerated while transferring larger volumes (Figure 5 and Figure 6). After further investigation, we realized that the water in the tip pulls on the air due to gravity, which causes the air to expand, making measuring with the graduations inaccurate. The graduations are intended to measure incompressible fluids within the syringe, while we were using them to measure air while under vacuum. The syringe graduations could not be expected to accurately indicate the volume of water pulled into the tip, because the air had expanded under vacuum. This explains the negative systematic error we were experiencing.

Our solution was to replace the built-in scale with a scale that accounted for the expansion. We compared expected volumes with actual measurements and noticed a linear relationship. We scaled up the graduations by a factor of 1:1.027 for both the 100–1000-μL, and 30–300-μL configurations. These adjusted scales are printed on paper or a transparency sheet and taped onto the syringe with the zero at the plunger when the pipette is in the latched position (scales appear in Appendix A: Figure A1 and Figure A2). With the new scale taped on the syringe, our validation testing met the ISO 8655 standard. Replacing the built-in scale with our scale corrected this effect. Our printed pipette with the adjusted scale meets ISO standards for accuracy and precision (Table 1 and Table 2).

## 4. Discussion and Conclusions

Our printed pipette improves on existing open design pipettes in several ways. Most significantly, the user is able to adjust the syringe accurately without verifying the volume with a scale. The pipette can be adjusted to any discrete volume within range. In addition, the assembly requires no permanent connections using tape or glue, which allows for re-configuration and easy replacement of parts. Conveniently, our design can also reach to the bottom of a 15-mL conical tube allowing tasks such as aspirating supernatant fluid from a cell pellet. Unlike existing designs, our design does not use a membrane that may wear out and require replacement. The only major limitation of this design compared to the biropipette [45] is the option for a pipette tip ejector. The biropipette also uses arguably easier to source parts (a pen vs. a syringe) relative to what is required for our design. The biropipette also was developed in OpenSCAD, which is free and open source CAD software, whereas our design was created in Solidworks, a proprietary software. This creates a limitation for users who may not have access to the proprietary software used to make the design. Fortunately, the produced STL files are universal and can be imported and modified by any CAD software.

The luer to pipette tip connection is a good candidate for a 3D-printed solution. Unfortunately, the current state of FDM cannot make this adapter with high enough resolution or, critically, surface smoothness [44]. Other printing techniques, such as stereolithography (SLA) and multijet printing (MJP), provide higher resolution and smoother finishing, yet FDM is the the most widely available and affordable of the printing technologies. SLA printers are coming down in price, but are still significantly more expensive, especially considering the higher cost of the consumable resin vs. FDM filament. Ultimately, the goal of this project is to make this pipette inexpensive and easy to make.

Our design relies heavily on the syringe, a specialized part, for the core function, as well as accuracy. Fortunately, as disposable syringes are a regulated medical device, they must meet high standards, making them a reliable part for this design. As good as the performance of our printed pipette is, a commercial pipette should be expected to be more reliable and preferred for critical procedures. Adjusting our printed pipette to the desired volume requires more time and attention, which would be tedious for procedures requiring many adjustments. On the other hand, due to the $6 cost to make it, many printed pipettes could be assembled and pre-set, each for a planned transfer, rather then re-adjusting one commercial pipette for each step. The printable pipette can also be used as a disposable, sparing the commercial micropipette when damage from volatile reagents is a concern or in situations were cross-contamination and resterilization is required. This pipette would also be ideal for high school and teaching labs or any resource-limited program.

The open design nature of this project encourages anyone to use and submit changes to improve and add features to this design. All of the working files and documentation are kept in a public repository [46]. Future directions for this project include developing a tip ejection system and additional configurations for transferring volumes below 30 μL, as well as increasing overall user friendliness, such as making the adjusting bolt easier to grip and improving ergonomics. It is our hope that this project will be under continuous development where improvements and compatibility with different syringes and printers can be crowd-sourced from community contributors.

## Figures and Tables

**Table 1:** ISO 8655 for 100–1000-μL comparing a commercial pipette with our printed pipette used with the existing 3-mL syringe scale and an adjusted scale.

		Mean	Systematic Error	% Sys. Err.	Random Error	% Rand. Err.
1000 μL	ISO 8655, 100–1000 μL	1000	8.00	0.80	3.00	0.30
	Commercial Pipette	1002.98	2.98	0.30	1.72	0.17
	Printed Pipette	949.29	−50.71	−5.07	0.60	0.06
	Printed Pipette Scale	1003.57	3.57	0.36	0.89	0.09
500 μL	ISO 8655, 100–1000 μL	500	8.00	1.60	3.00	0.60
	Commercial Pipette	503.67	3.67	0.73	0.49	0.10
	Printed Pipette	475.99	−24.01	−4.80	4.75	1.00
	Printed Pipette Scale	503.62	3.62	0.72	1.64	0.33
200 μL	ISO 8655, 100–1000 μL	200	8.00	4.00	3.00	1.50
	Commercial Pipette	204.61	4.61	2.30	0.15	0.07
	Printed Pipette	186.55	−13.45	−6.72	1.31	0.70
	Printed Pipette Scale	201.87	1.87	0.94	1.47	0.73
100 μL	ISO 8655, 100–1000 μL	100	8.00	8.00	3.00	3.00
	Commercial Pipette	104.29	4.29	4.29	1.65	1.58
	Printed Pipette	94.02	−5.98	−5.98	4.81	5.12
	Printed Pipette Scale	101.00	1.00	1.00	1.05	1.04

**Table 2:** ISO 8655 for 30–300-μL comparing a commercial pipette with our printed pipette used with the existing 3-mL syringe scale and an adjusted scale.

		Mean	Systematic Error	% Sys. Err.	Random Error	% Rand. Err.
300 μL	ISO 8655, 30–300 μL	300	4.00	1.33	1.50	0.50
	Commercial Pipette	301.19	1.19	0.40	0.53	0.18
	Printed Pipette	286.91	−13.09	−4.36	0.42	0.15
	Printed Pipette Scale	299.11	−0.89	−0.30	0.48	0.16
200 μL	ISO 8655, 30–300 μL	200	4	2	1.5	0.75
	Commercial Pipette	200.06	0.06	0.03	0.46	0.23
	Printed Pipette	193.40	−6.60	−3.30	2.86	1.48
	Printed Pipette Scale	200.57	0.57	0.28	0.86	0.43
50 μL	ISO 8655, 30–300 μL	50	4	8	1.5	3
	Commercial Pipette	49.02	−0.98	−1.96	0.10	0.20
	Printed Pipette	49.62	−0.38	−0.76	1.26	2.53
	Printed Pipette Scale	48.73	−1.27	−2.54	1.11	2.27
30 μL	ISO 8655, 30–300 μL	30	4	13.3	1.5	5
	Commercial Pipette	29.08	−0.92	−3.06	0.09	0.31
	Printed Pipette	29.22	−0.78	−2.59	0.31	1.07
	Printed Pipette Scale	27.78	−2.22	−7.41	1.37	4.93

## Supplementary Materials

The following are available online at https://zenodo.org/record/1220094#.WtcQ2FKSCJ1: File S1: body-clip.STL, File S2: plunger.STL, Video S1: Assembly of the pipette, Video S2: Operation of the pipette.

## Appendix A. Additional Materials

**Table A1:** Parts and cost for the 30–300-μL pipette.

30–300 mL Configuration:	Source	Unit Price	Part Number
Filament	Makerbot	$1.63	NA
1-mL Syringe	BD Biosciences	$0.15	309628
M3 Bolt, 35 mm	McMaster-Carr	$0.12	91287A026
M3 Nut	McMaster-Carr	$0.01	90591A121
Music Wire Compression Springs * (2)	Jones Spring Co	$1.23	C10-022-048
Female Luer to 1/8" Hose Barb Adapter	Cole-Parmer	$0.40	EW-30800-08
Tygon Tubing, 1/16" ID × 3/16" OD	Cole-Parmer	$0.03	EW-06407-72
300-μL Tips (10)	Fisher Scientific	$0.34	02-707-447
	Total	$3.91	

* Spring specifications: Overall length 38 mm, OD 7.62 mm, wire diameter 0.56 mm, Max load 0.86 kg, with closed and flat ends. Also available from McMaster-Carr P/N: 9657K347.

**Table A2:** Parts and cost for the 100–1000-μL pipette.

100–1000 mL Configuration:	Source	Unit Price	Part Number
Filament	Makerbot	$1.63	NA
3-mL Syringe	BD Biosciences	$0.73	309657
M3 Bolt, 35 mm	McMaster-Carr	$0.12	91287A026
M3 Nut	McMaster-Carr	$0.01	90591A121
Music Wire Compression Springs * (2)	Jones Spring Co	$1.23	C10-022-048
Female luer to 5/32" Hose Barb Adapter	Cole-Parmer	$0.66	EW-45508-06
Tygon Tubing, 1/8" ID × 1/4" OD	Cole-Parmer	$0.05	EW-06407-76
1000-μL Tips (10)	Fisher Scientific	$0.41	02-707-400
	Total	$4.84	

* Spring specifications: Overall length 38 mm, OD 7.62 mm, wire diameter 0.56 mm, Max load 0.86 kg, with closed and flat ends. Also available from McMaster-Carr P/N: 9657K347.
